# The genome sequence of the Ashy Button, *Acleris sparsana *(Denis & Schiffermüller, 1775)

**DOI:** 10.12688/wellcomeopenres.19533.1

**Published:** 2023-06-12

**Authors:** Douglas Boyes, David C. Lees, James Hammond, University of Oxford and Wytham Woods Genome Acquisition Lab, Natural History Museum Genome Acquisition Lab, Darwin Tree of Life Barcoding collective, Wellcome Sanger Institute Tree of Life programme, Wellcome Sanger Institute Scientific Operations: DNA Pipelines collective, Tree of Life Core Informatics collective, Darwin Tree of Life Consortium

**Affiliations:** 1 UK Centre for Ecology & Hydrology, Wallingford, England, UK; 2 Natural History Museum, London, England, UK; 3 University of Oxford, Oxford, England, UK

**Keywords:** Acleris sparsana, Ashy Button, genome sequence, chromosomal, Lepidoptera

## Abstract

We present a genome assembly from an individual male *Acleris sparsana* (the Ashy Button; Arthropoda; Insecta; Lepidoptera; Tortricidae). The genome sequence is 589.5 megabases in span. Most of the assembly is scaffolded into 30 chromosomal pseudomolecules, including the Z sex chromosome. The mitochondrial genome has also been assembled and is 16.4 kilobases in length. Gene annotation of this assembly on Ensembl identified 22,123 protein coding genes.

## Species taxonomy

Eukaryota; Opisthokonta; Metazoa; Eumetazoa; Bilateria; Protostomia; Ecdysozoa; Panarthropoda; Arthropoda; Mandibulata; Pancrustacea; Hexapoda; Insecta; Dicondylia; Pterygota; Neoptera; Endopterygota; Amphiesmenoptera; Lepidoptera; Glossata; Neolepidoptera; Heteroneura; Ditrysia; Apoditrysia; Tortricoidea; Tortricidae; Tortricinae; Tortricini; *Acleris*, *Acleris sparsana* (Denis & Schiffermüller, 1775) (NCBI:txid758717).

## Background

The Ashy Button *Acleris sparsana* (Denis & Schiffermüller, 1775) is a moth in the Tortricidae family. The species’ vernacular name is a reference to the ash-grey colour of the forewing. Like other members of its genus, the species is polymorphic, and three colour forms are recognised from Britain and Ireland ( Bradley *et al.*, 1973). *Acleris sparsana* is found across the islands of the North Atlantic, and is widespread across north and central Europe ( GBIF Secretariat, 2022). The species is also recorded south to the Mediterranean and east to the Black Sea ( Bradley *et al.*, 1973; GBIF Secretariat, 2022).

The larva feeds from June to August between spun leaves of the foodplant. The preferred foodplants in Britain and Ireland are beech ( *Fagus*), hornbeam ( *Carpinus*), and sycamore ( *Acer pseudoplatanus*), but elsewhere the larvae have been recorded feeding on *Sorbus*, *Betula*, *Quercus*, *Populus* and *Rubus* ( Bradley *et al.*, 1973; Elliott *et al.*, 2018). Adults occur from August and overwinter, flying until the following May ( Bradley *et al.*, 1973; Elliott *et al.*, 2018). Adults come to light and are attracted to sugar and ivy blossom ( Bradley *et al.*, 1973).

The genome of the ashy button, *Acleris sparsana*, was sequenced as part of the Darwin Tree of Life Project, a collaborative effort to sequence all named eukaryotic species in the Atlantic Archipelago of Britain and Ireland. Here we present a chromosomally complete genome sequence for *Acleris sparsana*, based on one male specimen from Wytham Woods, Oxfordshire.

## Genome sequence report

**Figure 1.**
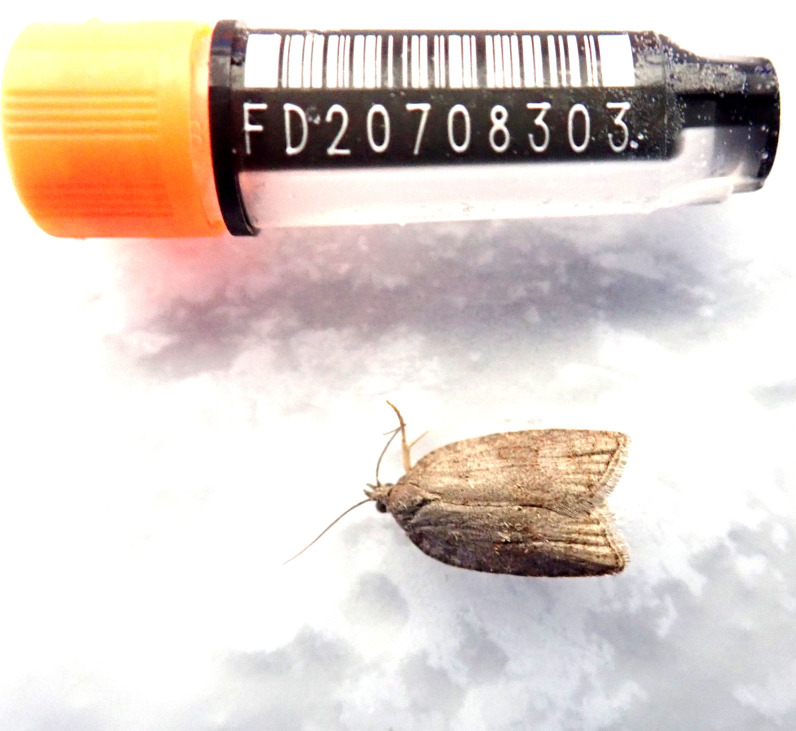
Photograph of the *Acleris sparsana* (ilAclSpar1) specimen used for genome sequencing.

The genome was sequenced from one male *Acleris sparsana* ( Figure 1) collected from Wytham Woods, Oxfordshire, UK (51.77, –1.34). A total of 37-fold coverage in Pacific Biosciences single-molecule HiFi long reads and 78-fold coverage in 10X Genomics read clouds were generated. Primary assembly contigs were scaffolded with chromosome conformation Hi-C data. Manual assembly curation corrected six missing joins or mis-joins, reducing the scaffold number by 5.71%.

**Figure 2.**
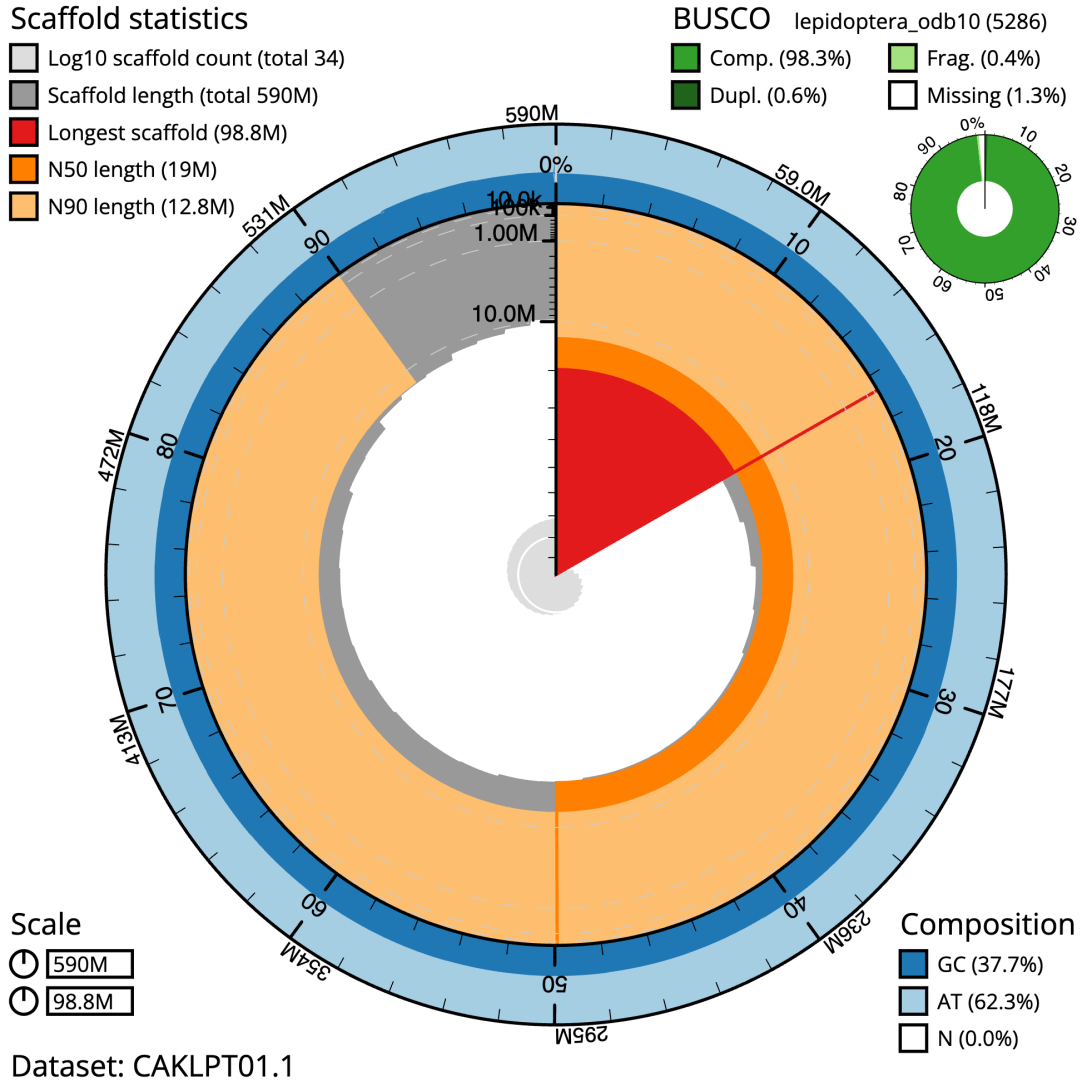
Genome assembly of *Acleris sparsana*, ilAclSpar1.1: metrics. The BlobToolKit Snailplot shows N50 metrics and BUSCO gene completeness. The main plot is divided into 1,000 size-ordered bins around the circumference with each bin representing 0.1% of the 589,546,063 bp assembly. The distribution of scaffold lengths is shown in dark grey with the plot radius scaled to the longest scaffold present in the assembly (98,792,858 bp, shown in red). Orange and pale-orange arcs show the N50 and N90 scaffold lengths (19,007,631 and 12,784,158 bp), respectively. The pale grey spiral shows the cumulative scaffold count on a log scale with white scale lines showing successive orders of magnitude. The blue and pale-blue area around the outside of the plot shows the distribution of GC, AT and N percentages in the same bins as the inner plot. A summary of complete, fragmented, duplicated and missing BUSCO genes in the lepidoptera_odb10 set is shown in the top right. An interactive version of this figure is available at https://blobtoolkit.genomehubs.org/view/ilAclSpar1.1/dataset/CAKLPT01.1/snail.

The final assembly has a total length of 589.5 Mb in 33 sequence scaffolds with a scaffold N50 of 19.0 Mb ( Table 1). Most (99.99%) of the assembly sequence was assigned to 30 chromosomal-level scaffolds, representing 29 autosomes and the Z sex chromosome. Chromosome-scale scaffolds confirmed by the Hi-C data are named in order of size ( Figure 2– Figure 5; Table 2). While not fully phased, the assembly deposited is of one haplotype. Contigs corresponding to the second haplotype have also been deposited. The mitochondrial genome was also assembled and can be found as a contig within the multifasta file of the genome submission.

**Table 1.** Genome data for *Acleris sparsana*, ilAclSpar1.1.

Project accession data		
Assembly identifier	ilAclSpar1.1	
Species	*Acleris sparsana*	
Specimen	ilAclSpar1	
NCBI taxonomy ID	758717.0	
BioProject	PRJEB47322	
BioSample ID	SAMEA8603209	
Isolate information	ilAclSpar1, male (DNA sequencing and Hi-C scaffolding) ilAclSpar2, male (RNA sequencing)	
Assembly metrics *		*Benchmark*
Consensus quality (QV)	57.9	*≥ 50*
*k*-mer completeness	100%	*≥ 95%*
BUSCO **	C:98.3%[S:97.7%,D:0.6%], F:0.4%,M:1.3%,n:5,286	*C ≥ 95%*
Percentage of assembly mapped to chromosomes	99.99%	*≥ 95%*
Sex chromosomes	Z chromosome	*localised homologous pairs*
Organelles	Mitochondrial genome assembled	*complete single alleles*
Raw data accessions		
PacificBiosciences SEQUEL II	ERR6808046	
10X Genomics Illumina	ERR6688755–ERR6688758	
Hi-C Illumina	ERR6688754	
PolyA RNA-Seq Illumina	ERR10377998	
Genome assembly		
Assembly accession	GCA_923062465.1	
*Accession of alternate haplotype*	GCA_923061745.1	
Span (Mb)	589.5	
Number of contigs	41	
Contig N50 length (Mb)	18.9	
Number of scaffolds	33	
Scaffold N50 length (Mb)	19.0	
Longest scaffold (Mb)	98.8	
Genome annotation		
Number of protein-coding genes	22,123	
Number of gene transcripts	22,280	

* Assembly metric benchmarks are adapted from column VGP-2020 of “Table 1: Proposed standards and metrics for defining genome assembly quality” from ( Rhie *et al.*, 2021). ** BUSCO scores based on the lepidoptera_odb10 BUSCO set using v5.3.2. C = complete [S = single copy, D = duplicated], F = fragmented, M = missing, n = number of orthologues in comparison. A full set of BUSCO scores is available at https://blobtoolkit.genomehubs.org/view/ilAclSpar1.1/dataset/CAKLPT01.1/busco.

**Figure 3.**
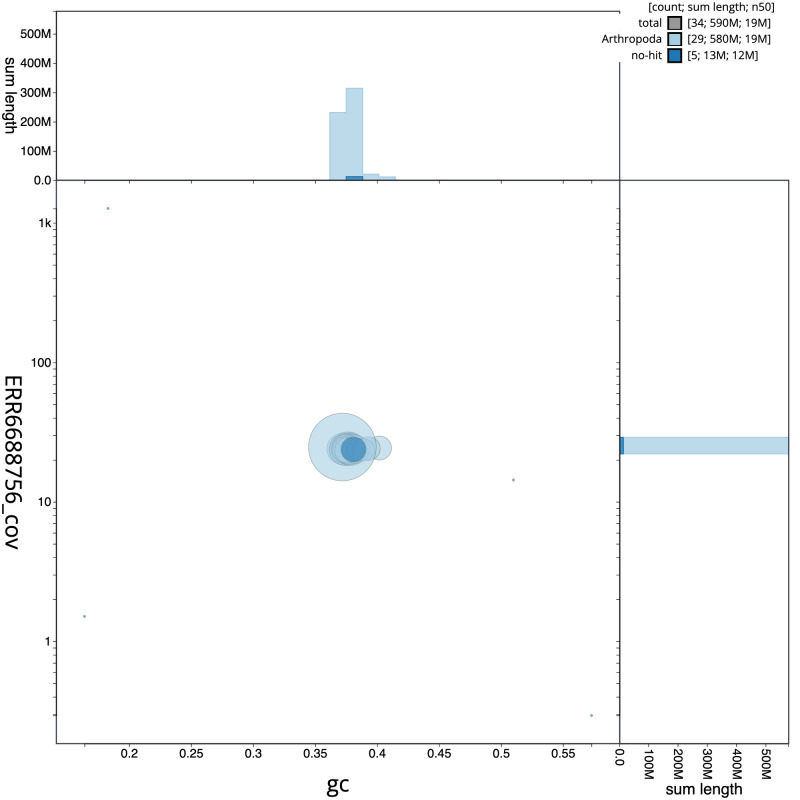
Genome assembly of *Acleris sparsana*, ilAclSpar1.1: BlobToolKit GC-coverage plot. Scaffolds are coloured by phylum. Circles are sized in proportion to scaffold length. Histograms show the distribution of scaffold length sum along each axis. An interactive version of this figure is available at https://blobtoolkit.genomehubs.org/view/ilAclSpar1.1/dataset/CAKLPT01.1/blob.

**Figure 4.**
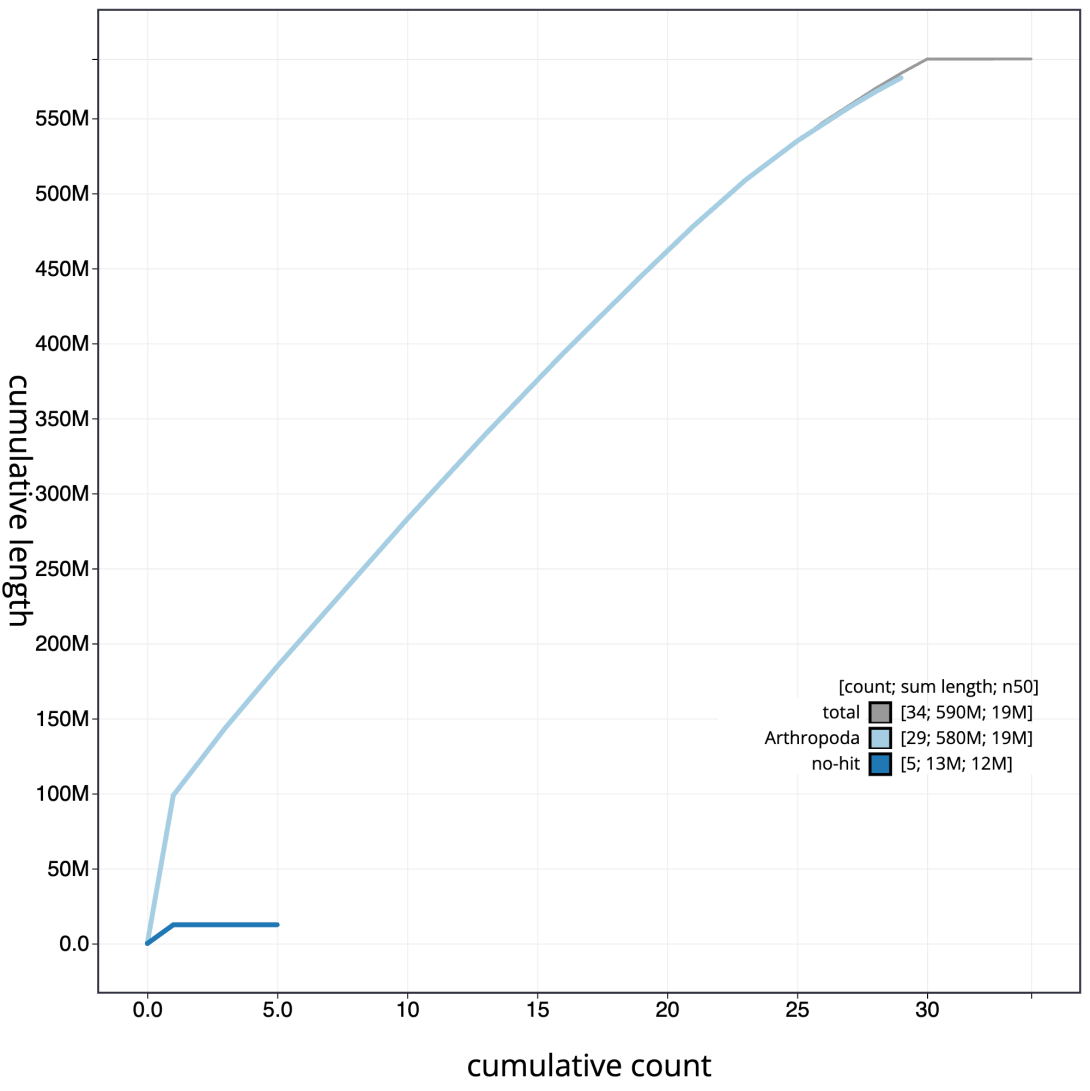
Genome assembly of *Acleris sparsana*, ilAclSpar1.1: BlobToolKit cumulative sequence plot. The grey line shows cumulative length for all scaffolds. Coloured lines show cumulative lengths of scaffolds assigned to each phylum using the buscogenes taxrule. An interactive version of this figure is available at https://blobtoolkit.genomehubs.org/view/ilAclSpar1.1/dataset/CAKLPT01.1/cumulative.

**Figure 5.**
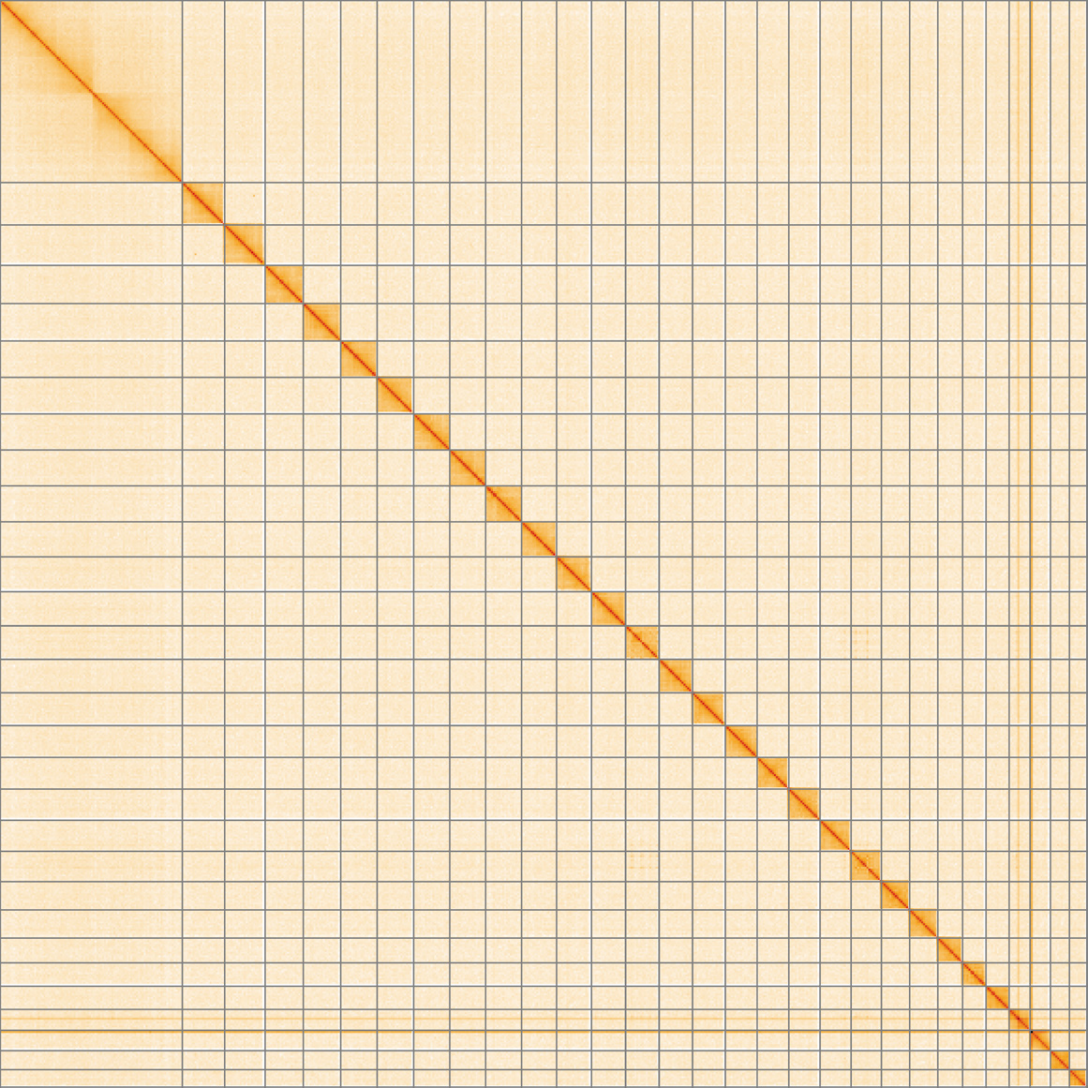
Genome assembly of *Acleris sparsana*, ilAclSpar1.1: Hi-C contact map of the ilAclSpar1.1 assembly, visualised using HiGlass. Chromosomes are shown in order of size from left to right and top to bottom. An interactive version of this figure may be viewed at https://genome-note-higlass.tol.sanger.ac.uk/l/?d=T9gi_l7NTdOpYx5SjkqwQQ.

**Table 2.** Chromosomal pseudomolecules in the genome assembly of *Acleris sparsana*, ilAclSpar1.

INSDC accession	Name	Length (Mb)	GC%
OV281286.1	1	22.96	37.5
OV281287.1	2	21.96	37.5
OV281288.1	3	20.75	37.5
OV281289.1	4	20.32	37.5
OV281290.1	5	19.79	38
OV281291.1	6	19.75	37.5
OV281292.1	7	19.59	37.5
OV281293.1	8	19.51	37
OV281294.1	9	19.48	37.5
OV281295.1	10	19.01	38
OV281296.1	11	18.94	37.5
OV281297.1	12	18.42	38
OV281298.1	13	18.24	38
OV281299.1	14	18.13	37.5
OV281300.1	15	17.75	38
OV281301.1	16	17.32	37.5
OV281302.1	17	17.12	37.5
OV281303.1	18	17.01	38
OV281304.1	19	16.74	38
OV281305.1	20	16.45	38
OV281306.1	21	15.44	38
OV281307.1	22	15.23	37.5
OV281308.1	23	13.39	37.5
OV281309.1	24	12.78	37.5
OV281310.1	25	12.5	38
OV281311.1	26	11.4	40
OV281312.1	27	11	39.5
OV281313.1	28	10.28	38.5
OV281314.1	29	9.45	39
OV281285.1	Z	98.79	37
OV281315.1	MT	0.02	18.5

The estimated Quality Value (QV) of the final assembly is 57.9 with *k*-mer completeness of 100%, and the assembly has a BUSCO v5.3.2 completeness of 98.3% (single = 97.7%, duplicated = 0.6%), using the lepidoptera_odb10 reference set ( *n* = 5,286).

Metadata for specimens, spectral estimates, sequencing runs, contaminants and pre-curation assembly statistics can be found at https://links.tol.sanger.ac.uk/species/758717.

## Genome annotation report

The *Acleris sparsana* genome assembly (GCA_923062465.1) was annotated using the Ensembl rapid annotation pipeline ( Table 1; https://rapid.ensembl.org/Acleris_sparsana_GCA_923062465.1/Info/Index). The resulting annotation includes 22,280 transcribed mRNAs from 22,123 protein-coding genes.

## Methods

### Sample acquisition and nucleic acid extraction

A male *Acleris sparsana* (specimen number Ox000978, ilAclSpar1) was collected from Wytham Woods, Oxfordshire (biological vice-county Berkshire), UK (latitude 51.77, longitude –1.34) on 2020-10-08. The specimen was taken from Woodland by Douglas Boyes (University of Oxford) using a light trap. The specimen was identified by the collector and snap-frozen on dry ice. This specimen was used for genome sequencing and Hi-C scaffolding. A second male specimen (specimen number NHMUK013698321, ilAclSpar2) was hand-picked by David Lees (Natural History Museum) in High Wycombe, UK (latitude 51.63, longitude –0.74) on 2021-10-10. This specimen was used for RNA sequencing.

DNA was extracted at the Tree of Life laboratory, Wellcome Sanger Institute (WSI). The ilAclSpar1 sample was weighed and dissected on dry ice with tissue set aside for Hi-C sequencing. Whole organism tissue was disrupted using a Nippi Powermasher fitted with a BioMasher pestle. High molecular weight (HMW) DNA was extracted using the Qiagen MagAttract HMW DNA extraction kit. Low molecular weight DNA was removed from a 20 ng aliquot of extracted DNA using the 0.8X AMpure XP purification kit prior to 10X Chromium sequencing; a minimum of 50 ng DNA was submitted for 10X sequencing. HMW DNA was sheared into an average fragment size of 12–20 kb in a Megaruptor 3 system with speed setting 30. Sheared DNA was purified by solid-phase reversible immobilisation using AMPure PB beads with a 1.8X ratio of beads to sample to remove the shorter fragments and concentrate the DNA sample. The concentration of the sheared and purified DNA was assessed using a Nanodrop spectrophotometer and Qubit Fluorometer and Qubit dsDNA High Sensitivity Assay kit. Fragment size distribution was evaluated by running the sample on the FemtoPulse system. 

RNA was extracted from head and thorax tissue of ilAclSpar2 in the Tree of Life Laboratory at the WSI using TRIzol, according to the manufacturer’s instructions. RNA was eluted in 50 μl RNAse-free water and its concentration assessed using a Nanodrop spectrophotometer and Qubit Fluorometer using the Qubit RNA Broad-Range (BR) Assay kit. Analysis of the integrity of the RNA was done using Agilent RNA 6000 Pico Kit and Eukaryotic Total RNA assay.

### Sequencing

Pacific Biosciences HiFi circular consensus and 10X Genomics read cloud DNA sequencing libraries were constructed according to the manufacturers’ instructions. Poly(A) RNA-Seq libraries were constructed using the NEB Ultra II RNA Library Prep kit. DNA and RNA sequencing were performed by the Scientific Operations core at the WSI on Pacific Biosciences SEQUEL II (HiFi), Illumina NovaSeq 6000 (RNA-Seq and 10X) instruments. Hi-C data were also generated from tissue of ilAclSpar1 using the Arimav2 kit and sequenced on the Illumina NovaSeq 6000 instrument.

### Genome assembly, curation and evaluation

Assembly was carried out with Hifiasm ( Cheng *et al.*, 2021) and haplotypic duplication was identified and removed with purge_dups ( Guan *et al.*, 2020). One round of polishing was performed by aligning 10X Genomics read data to the assembly with Long Ranger ALIGN, calling variants with FreeBayes ( Garrison & Marth, 2012). The assembly was then scaffolded with Hi-C data ( Rao *et al.*, 2014) using SALSA2 ( Ghurye *et al.*, 2019). The assembly was checked for contamination as described previously ( Howe *et al.*, 2021). Manual curation was performed using HiGlass ( Kerpedjiev *et al.*, 2018) and Pretext ( Harry, 2022). The mitochondrial genome was assembled using MitoHiFi ( Uliano-Silva *et al.*, 2022), which runs MitoFinder ( Allio *et al.*, 2020) or MITOS ( Bernt *et al.*, 2013) and uses these annotations to select the final mitochondrial contig and to ensure the general quality of the sequence.

A Hi-C map for the final assembly was produced using bwa-mem2 ( Vasimuddin *et al.*, 2019) in the Cooler file format ( Abdennur & Mirny, 2020). To assess the assembly metrics, the *k*-mer completeness and QV consensus quality values were calculated in Merqury ( Rhie *et al.*, 2020). This work was done using Nextflow ( Di Tommaso *et al.*, 2017) DSL2 pipelines “sanger-tol/readmapping” ( Surana *et al.*, 2023a) and “sanger-tol/genomenote” ( Surana *et al.*, 2023b). The genome was analysed within the BlobToolKit environment ( Challis *et al.*, 2020) and BUSCO scores ( Manni *et al.*, 2021; Simão *et al.*, 2015) were calculated.

Table 3 contains a list of relevant software tool versions and sources.

**Table 3.** Software tools: versions and sources.

Software tool	Version	Source
BlobToolKit	4.0.7	https://github.com/blobtoolkit/blobtoolkit
BUSCO	5.3.2	https://gitlab.com/ezlab/busco
FreeBayes	1.3.1-17-gaa2ace8	https://github.com/freebayes/freebayes
Hifiasm	0.15.3	https://github.com/chhylp123/hifiasm
HiGlass	1.11.6	https://github.com/higlass/higlass
Long Ranger ALIGN	2.2.2	https://support.10xgenomics.com/genome-exome/ software/pipelines/latest/advanced/other-pipelines
Merqury	MerquryFK	https://github.com/thegenemyers/MERQURY.FK
MitoHiFi	2	https://github.com/marcelauliano/MitoHiFi
PretextView	0.2	https://github.com/wtsi-hpag/PretextView
purge_dups	1.2.3	https://github.com/dfguan/purge_dups
SALSA	2.2	https://github.com/salsa-rs/salsa
sanger-tol/genomenote	v1.0	https://github.com/sanger-tol/genomenote
sanger-tol/readmapping	1.1.0	https://github.com/sanger-tol/readmapping/tree/1.1.0

### Genome annotation

The BRAKER2 pipeline ( Brůna *et al.*, 2021) was used in the default protein mode to generate annotation for the *Acleris sparsana* assembly (GCA_923062465.1). in Ensembl Rapid Release.

### Wellcome Sanger Institute – Legal and Governance

The materials that have contributed to this genome note have been supplied by a Darwin Tree of Life Partner. The submission of materials by a Darwin Tree of Life Partner is subject to the **‘Darwin Tree of Life Project Sampling Code of Practice’**, which can be found in full on the Darwin Tree of Life website here. By agreeing with and signing up to the Sampling Code of Practice, the Darwin Tree of Life Partner agrees they will meet the legal and ethical requirements and standards set out within this document in respect of all samples acquired for, and supplied to, the Darwin Tree of Life Project.

Further, the Wellcome Sanger Institute employs a process whereby due diligence is carried out proportionate to the nature of the materials themselves, and the circumstances under which they have been/are to be collected and provided for use. The purpose of this is to address and mitigate any potential legal and/or ethical implications of receipt and use of the materials as part of the research project, and to ensure that in doing so we align with best practice wherever possible. The overarching areas of consideration are:

- Ethical review of provenance and sourcing of the material
- Legality of collection, transfer and use (national and international)

Each transfer of samples is further undertaken according to a Research Collaboration Agreement or Material Transfer Agreement entered into by the Darwin Tree of Life Partner, Genome Research Limited (operating as the Wellcome Sanger Institute), and in some circumstances other Darwin Tree of Life collaborators.

## Data Availability

European Nucleotide Archive: *Acleris sparsana* (ashy button). Accession number PRJEB47322; https://identifiers.org/ena.embl/PRJEB47322. ( Wellcome Sanger Institute, 2021) The genome sequence is released openly for reuse. The *Acleris sparsana* genome sequencing initiative is part of the Darwin Tree of Life (DToL) project. All raw sequence data and the assembly have been deposited in INSDC databases. Raw data and assembly accession identifiers are reported in Table 1.

## Author information

Members of the University of Oxford and Wytham Woods Genome Acquisition Lab are listed here: https://doi.org/10.5281/zenodo.4789928.

Members of the Natural History Museum Genome Acquisition Lab are listed here: https://doi.org/10.5281/zenodo.4790042.

Members of the Darwin Tree of Life Barcoding collective are listed here: https://doi.org/10.5281/zenodo.4893703.

Members of the Wellcome Sanger Institute Tree of Life programme are listed here: https://doi.org/10.5281/zenodo.4783585.

Members of Wellcome Sanger Institute Scientific Operations: DNA Pipelines collective are listed here: https://doi.org/10.5281/zenodo.4790455.

Members of the Tree of Life Core Informatics collective are listed here: https://doi.org/10.5281/zenodo.5013541.

Members of the Darwin Tree of Life Consortium are listed here: https://doi.org/10.5281/zenodo.4783558.
