# Supplementary material for: The genome sequence of the Ashy Button, Acleris sparsana (Denis & Schiffermüller, 1775)
Source: Wellcome Open Res. 2023 Jun 12;8:241. [Version 1] doi: 10.12688/wellcomeopenres.19533.1 (PMC10907877; doi:10.12688/wellcomeopenres.19533.1)
Supplement: Peer review history

# Reviewer report: Reviewer response for version 1

**DOI:** 10.21956/wellcomeopenres.21637.r74500

**Published:** 2024-03-01

**Authors:** Thayná Lopes

**Affiliations:** 1 Universidade Estadual de Londrina, Londrina, State of Paraná, Brazil

This genome note reports a high-quality assembly and annotation of the genome of Ashy Button, *Acleris sparsana, *a moth in the Tortricidae family. 

 The methods that were used are perfectly appropriate for this assembly, and the resulting genome is of excellent quality and will be an amazing resource for moth researchers in genomics, evolutionary biology, and entomology.

 The article provides structured information on the sequencing and assembly methods, and database identifier citations.

 I believe it would be interesting to add more information about the species. Explaining the relevance of this species and why generating data for this type of insect would add to both summary and background sections.

Are sufficient details of methods and materials provided to allow replication by others?

Yes

Is the rationale for creating the dataset(s) clearly described?

Yes

Are the datasets clearly presented in a useable and accessible format?

Yes

Are the protocols appropriate and is the work technically sound?

Yes

Reviewer Expertise:

Bioinformatics (genomics, proteomics and transcriptomics) and molecular genetics of insects

I confirm that I have read this submission and believe that I have an appropriate level of expertise to confirm that it is of an acceptable scientific standard.

# Reviewer report: Reviewer response for version 1

**DOI:** 10.21956/wellcomeopenres.21637.r74501

**Published:** 2024-02-29

**Authors:** Jason Hill

**Affiliations:** 1 Science for Life Laboratory, Department of Medical Biochemistry and Microbiology, Uppsala University, Uppsala, Sweden

The authors assembled and annotated the genome of the Ashy Button moth, *Acleris sparsana. *The methods described in the process represent the current best practices for assembling and annotating Lepidoptera genomes and the result is a highly contiguous chromosome level assembly and well represented gene annotation. This addition to the growing set of high-quality Lepidoptera annotations is welcome to the community and will aid in species specific and comparative genomics research.

Are sufficient details of methods and materials provided to allow replication by others?

Yes

Is the rationale for creating the dataset(s) clearly described?

Yes

Are the datasets clearly presented in a useable and accessible format?

Yes

Are the protocols appropriate and is the work technically sound?

Yes

Reviewer Expertise:

Population genomics, genome assembly, Lepidoptera biology.

I confirm that I have read this submission and believe that I have an appropriate level of expertise to confirm that it is of an acceptable scientific standard.
